# Supplementary material for: The Hippo pathway terminal effector TAZ/WWTR1 mediates oxaliplatin sensitivity in p53 proficient colon cancer cells
Source: BMC Cancer. 2024 May 14;24:587. doi: 10.1186/s12885-024-12316-4 (PMC11092100; doi:10.1186/s12885-024-12316-4)
Supplement: Supplementary file 1 — Supplementary Material 1. [file 12885_2024_12316_MOESM1_ESM.pdf]

SUPPLEMENTARY MATERIAL

The Hippo pathway terminal effector TAZ/WWTR1 mediates oxaliplatin sensitivity in p53 proficient colon cancer cells

Věra Slaninová, Lisa Heron-Milhavet, Mathilde Robin, Laura Jeanson, Adam Aissanou, Diala Kantar, Diego Tosi, Laurent Bréhélin, Céline Gongora, and Alexandre Djiane

Slaninova et al. Figure S1

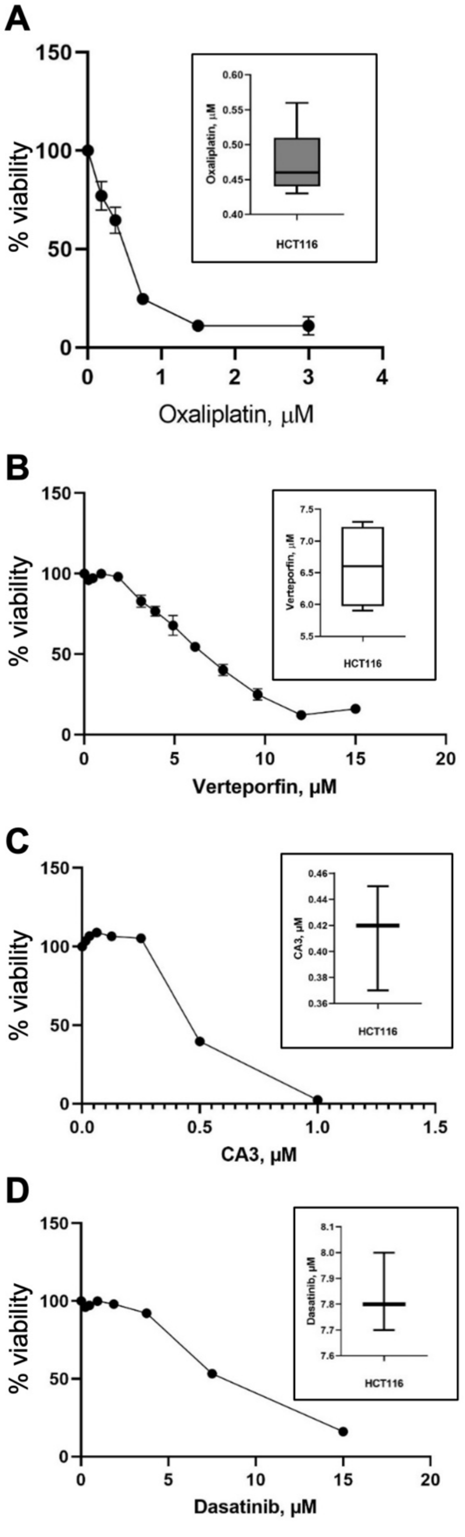

Supplemental Figure S1. Cell viability in response to drugs

Cells were treated with increasing doses of oxaliplatin (**A**), Verteporfin (**B**), CA3 (**C**) and Dasatinib (**D**) for 96h. Cell viability analysis was then assessed using SRB assay and the IC50 of each drug could be calculated as the concentration that reduced cell numbers by 50% (shown in the insets).

Slaninova et al. Figure S2

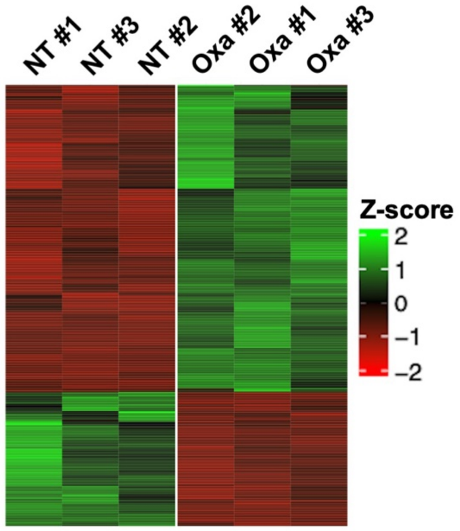

Supplemental Figure S2. Genes regulated by oxaliplatin treatment
Heat map corresponding to the genes differentially expressed in HCT116 cells after 24h of oxaliplatin treatment at IC50 (see Supplemental Table 1). The three replicates for the non-treated (NT) and treated (Oxa) are shown.

Slaninova et al. Figure S3

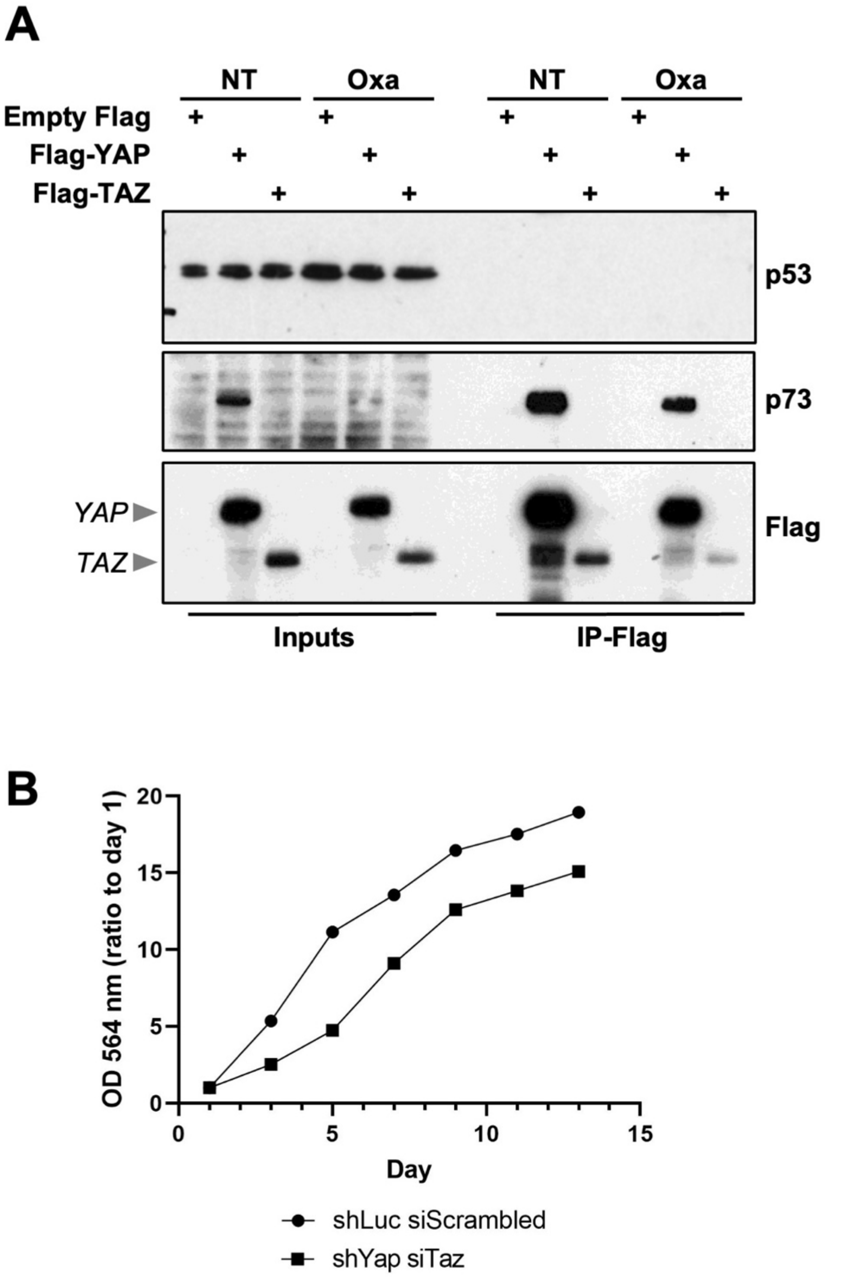

Supplemental Figure S3.

A. Interaction between YAP/TAZ and p53 protein family members in HCT116 cells

Western blot analysis of Flag (YAP or TAZ) immunoprecipitates on protein extracts from HCT116 treated (Oxa), or not (NT) showing an interaction between YAP and p73 (n=3).

B. Effect of shYAP/siTAZ on the growth of HCT116 cells

Proliferation curve measured by colorimetric SRB assay

Figure 1C

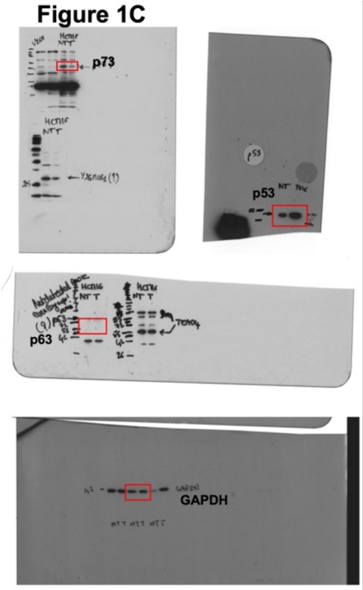

Figure 2B

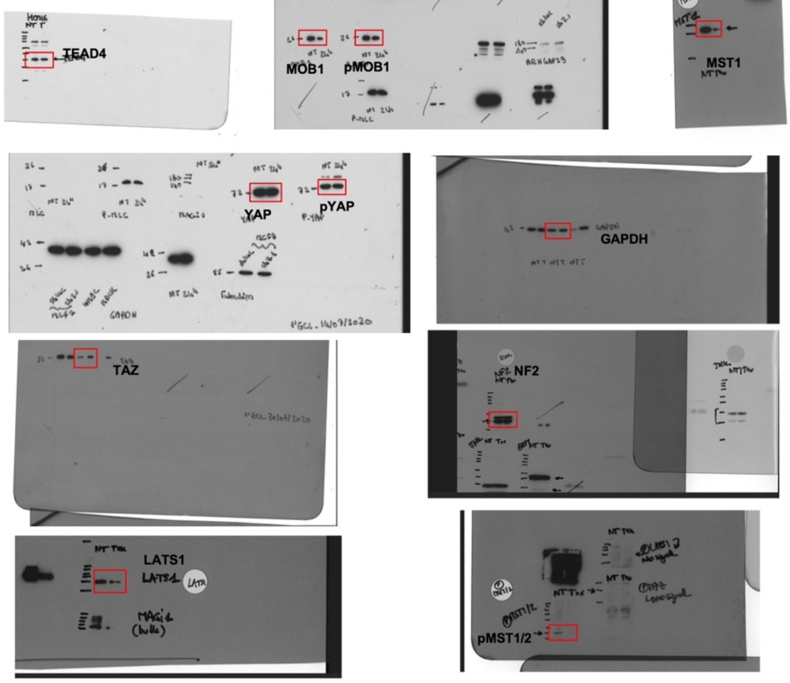

Figure 2C

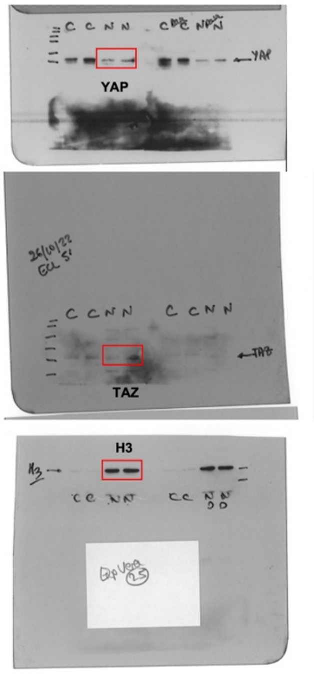

Figure 3C

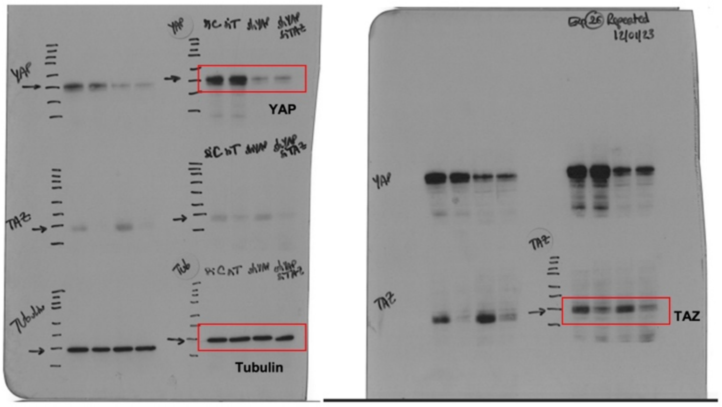

Supplemental Figure S4. Uncropped western-blots - part 1

Figure 3E

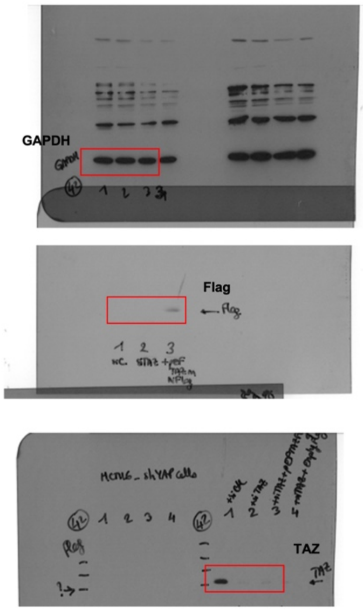

Figure 6A

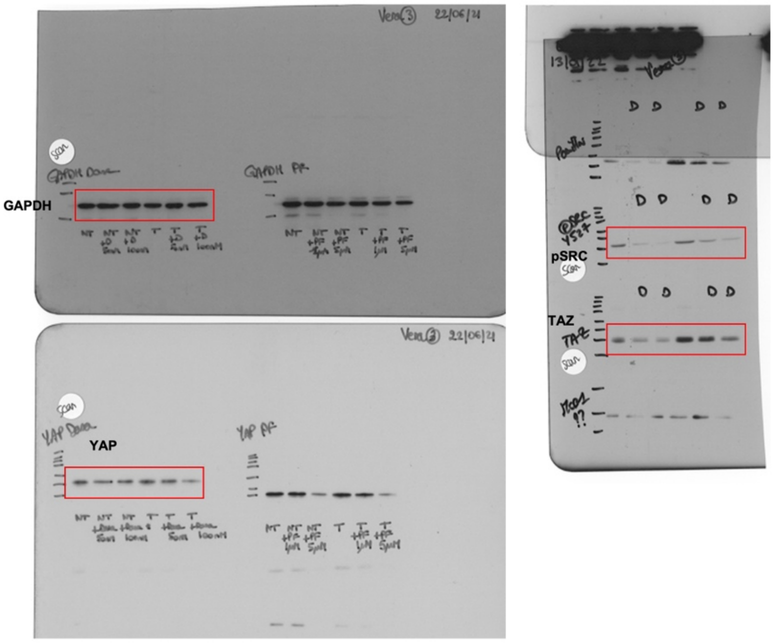

Figure S3A

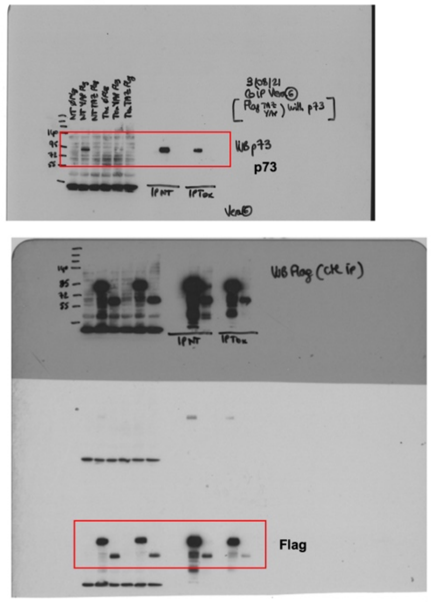

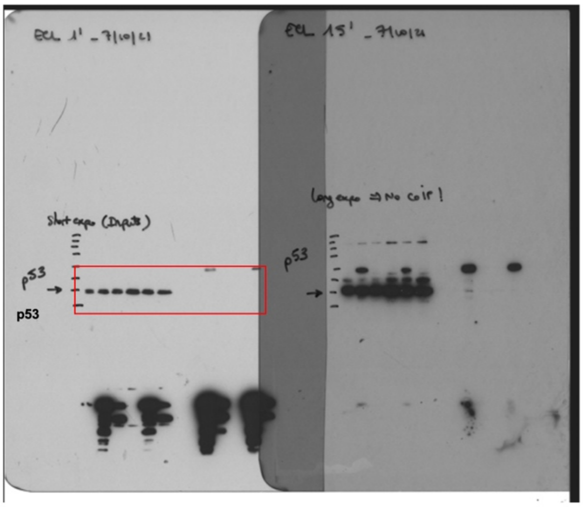

Supplemental Figure S5. Uncropped western-blots - part 2

Supplemental Table 1. Differentially expressed genes in HCT116 cells after oxaliplatin treatment for 24h at IC50

Only differentially expressed genes with an adjusted p-value< 0.05 are shown. Columns are:
test_id: position and description of the feature tested
ENSG_ID: Ensemble gene ID
symbol: gene symbol
sample_1: first group in the comparison; untreated cells
sample_2: second group in the comparison; cells treated with oxaliplatin
mean_1: mean of normalized count for the first group in the comparison
mean_2: mean of normalized count for the second group in the comparison
log2FoldChange: log2 fold change estimates
pvalue: pvalue
padj: pvalue adjusted for multiple testing with the Benjamini-Hochberg procedure, which controls false discovery rate (FDR)

Supplemental Table 2. g:profiler functional enrichment analyses

Columns are :
GO_ID: Gene Ontology
KEGG_ID: KEGG pathways
REAC_ID: Reactome pathways
WP_ID: WikiPathways
TF_ID: regulatory motif matches from TRANSFAC
MIRNA_ID: miRNA targets from miRTarBase
CORUM_ID: protein complexes from CORUM
HPA_ID: tissue specificity from Human Protein Atlas
HP_ID: human disease phenotypes from Human Phenotype Ontology
Description: description of the functional group
p.Val: p-value
FDR: false discovery rate
Genes: genes found in the intersection
